# Performance of Swedish Warmblood fragile foal syndrome carriers and breeding prospects

**DOI:** 10.1186/s12711-021-00693-4

**Published:** 2022-01-21

**Authors:** Michela Ablondi, Martin Johnsson, Susanne Eriksson, Alberto Sabbioni, Åsa Gelinder Viklund, Sofia Mikko

**Affiliations:** 1 Department of Veterinary Science, Università degli Studi di Parma, 43126 Parma, Italy; 2 Dept. of Animal Breeding and Genetics, Swedish University of Agricultural Sciences, PO Box 7023, S-750 07 Uppsala, Sweden

## Abstract

**Background:**

Warmblood fragile foal syndrome (WFFS) is a monogenetic defect caused by a recessive lethal missense point mutation in the *procollagen‐lysine, 2‐oxoglutarate 5‐dioxygenase 1* gene (*PLOD1*, c.2032G>A). The majority of homozygous WFFS horses are aborted during gestation. Clinical signs of affected horses include fragile skin, skin and mucosa lacerations, hyperextension of the articulations, and hematomas. In spite of its harmful effect, a relatively high frequency of WFFS carriers has been found in Warmblood horses, suggesting a heterozygote advantage. Thus, in this study our aims were to: (1) estimate the frequency of WFFS carriers in the Swedish Warmblood breed (SWB), (2) estimate the effect of WFFS carrier genotype on performance traits in two SWB subpopulations bred for different disciplines, and (3) simulate the potential effects of balancing selection and different selection strategies on the frequency of carriers.

**Methods:**

In total, 2288 SWB sport horses born between 1971 and 2020 were tested for the WFFS mutation and had estimated breeding values (EBV) for ten traditional evaluating and 50 linear descriptive traits.

**Results:**

The frequency of WFFS carriers calculated from a pool of 511 randomly selected SWB horses born in 2017 was equal to 7.4% and ranged from 0.0 to 12.0% among the whole set of tested SWB horses, starting from 1971 till 2020. The effect of the WFFS carrier genotype was significant for several EBV mainly related to movements and dressage traits and especially for horses not bred for the show jumping discipline. Using simulation, we showed that balancing selection can maintain a recessive lethal allele in populations such as the SWB breed over generations and that the frequency is expected to slowly decrease in absence of balancing selection. Finally, we showed that selection against carrier sires can result in a more rapid decrease of the frequency of the mutant allele over time.

**Conclusion:**

Further research is needed to confirm the apparent association between equine performance and the WFFS carrier genotype. Identification of such associations or new causative mutations for horse performance traits can serve as new tools in horse breeding to select for healthy, sustainable, and better performing horses.

**Supplementary Information:**

The online version contains supplementary material available at 10.1186/s12711-021-00693-4.

## Background

Warmblood fragile foal syndrome (WFFS) is an autosomal monogenetic disease that has raised much concern among warmblood horse breeders in recent years due to its negative effects on foal and foetus survival [1], with impacts on both animal welfare and the economy of horse breeders. WFFS is caused by a recessive lethal missense point mutation in the *procollagen‐lysine, 2‐oxoglutarate 5‐dioxygenase 1* gene (*PLOD1*, c.2032G>A), which encodes the enzyme lysyl hydroxylase (LH1), that acts on collagen biosynthesis by hydroxylation of lysyl residues in collagens. A deficit in LH1 causes mechanical instability of the affected tissues due to the reduction in collagen crosslinks [2]. The majority of WFFS recessive homozygous foals are assumed to be lost by abortion during late gestation. The liveborn affected foals show severe skin fragility, resulting in open lesions and joint hyper-elasticity, and they often need to be euthanized shortly after birth since they are not viable [3, 4].

Previous analyses based on pedigree data suggested segregation of the *PLOD1* lethal allele in several European Warmblood horse breeds, including the Hanoverian, Selle Français, KWPN, Oldenburg, and Westphalian studbooks [5]. In spite of the harmful effect of the mutation in the homozygous state, the frequency of WFFS carriers is relatively high in Warmblood breeds. Recent studies reported frequencies of WFFS carriers ranging from 11 to 30% among several Warmblood breeds [6–8]. In contrast, a much lower frequency of 2.4% was observed among 716 tested Thoroughbreds [9]. The lethal WFFS allele has not been detected in most non-warmblood breeds, which supports the exclusivity of the WFFS mutation to Warmbloods and Thoroughbred horses [6, 9]. Nevertheless, a recent study found a few WFFS carriers in Paint, Quarter, and Haflinger horses [10].

Warmblood sport horses are strongly selected for performance, mainly for the dressage and show jumping disciplines. Several of the traits under selection favor a high degree of joint mobility, smooth and supple movements, and elastic gaits in dressage horses, and extreme athleticism and power in show jumping horses. The clinical characteristics of hypermobility in horses are not comprehensively described, but there is a consensus that some horses are extremely flexible, supple, or even hypermobile. In humans, hypermobility is classified as a disease, although people with hypermobility spectrum disorders often outperform in sports that demand high levels of mobility [11]. Considering the known deleterious effect of the WFFS allele on the biosynthesis of collagen, and the joint hyper-elasticity seen in homozygotes for the WFFS allele, we hypothesize that it might also have an effect on flexibility and thus gait quality in carriers.

The WFFS allele has been reported to be associated with estimated breeding values (EBV) for conformation, gaits, and dressage traits in warmblood horses [6]. Therefore, balancing selection may have influenced the frequency of WFFS carriers in warmblood sport horse populations, including the Swedish Warmblood breed (SWB), which is selected for equestrian sport purposes [12]. Ablondi et al. [12] revealed a genetic divergence among SWB horses because of their specialization in different disciplines, by comparing two subgroups of SWB with high and low EBV for show jumping, which reflects selection for show jumping and dressage disciplines, respectively.

There are several examples of recessive deleterious or lethal alleles in domestic animals that appear to have been driven to high frequency by balancing selection. If a detrimental allele also has a favorable effect on a trait under selection or is in tight linkage disequilibrium with such a favorable variant, its frequency may increase in spite of its deleterious effect on survival or fertility. This is the case with a series of alleles in dairy cattle [13–15], beef cattle [16–18], and pigs [19, 20]. While it is possible for any allele to increase in frequency due to genetic drift, a recessive lethal allele is unlikely to become common by genetic drift alone, even in a small population [21].

A genetic test for WFFS is available and is currently used in the SWB to avoid mating between carriers. This policy improves animal welfare by eliminating the risk of affected foals. However, this genetic test could also be used for marker-assisted selection against the WFFS allele. The outcomes and effectiveness of such selection depend on its implementation in the breeding scheme and on whether the frequency of WFFS carriers is driven by balancing selection. Based on the economic and welfare relevance of WFFS, together with the suspected balancing selection on this deleterious allele in Warmblood breeds, our aims were to: (1) estimate the frequency of WFFS carriers in the SWB breed, (2) estimate the effect of the genotype of the WFFS carriers on performance traits, with a specific focus on the two SWB subpopulations that were previously reported to reflect horses selected for two different sport disciplines [12], and (3) simulate the potential effects of balancing selection and different selection strategies on expected future changes in carrier frequency.

## Methods

### Data description

Genotype data from 2288 sport horses (1216 males and 1072 females) born between 1971 and 2020 were analyzed in this study. To evaluate the frequency of WFFS carriers in the current generation of SWB horses, 511 randomly selected horses born in 2017, i.e., before the genetic test for WFFS became widely used, were genotyped specifically for this purpose. In addition, 380 previously analyzed SWB horses [12, 22] were genotyped for WFFS. More information on the selection of those 380 horses was reported in a previous study [22]. The WFFS genotypes of the remaining 1397 horses included in our study were provided by the Animal Genetics Laboratory, Swedish University of Agricultural Sciences, through routine service available for the breeders. These 1397 horses included mandatory genotyping of breeding stallions and breeder-based choices of mares and offspring to be genotyped.

Estimated breeding values (EBV) from the routine genetic evaluation in 2020 were available for all the 2288 genotyped horses included in the study. The available traits can be divided into two categories: (1) traditional evaluating traits and (2) linear descriptive traits. The evaluating traits are related to the breeding objective and their EBV are obtained with multi-trait animal models based on data from tests and competition results of young horses. The ten evaluating traits included: type, correctness of legs, walk, trot, canter, temperament for gaits, jumping technique, jumping temperament, show jumping and dressage [23]. Fifty linear descriptive traits were available and their EBV were obtained with univariate animal models based on descriptive assessments from tests on young horses that were described from one biological extreme to the other [24]. Of the 50 linear descriptive traits, 13 were related to jumping, 15 to movements, 21 described the horse’s conformation, and one was a behavior trait. To account for different reliabilities of the EBV, we first removed all horses that had only parental average (PA) EBV. Then, we obtained de-regressed EBV (DEBV) from the EBV and reliabilities for animals and parents, using the DRP package (https://rdrr.io/github/camult/DRP/) in R [25] based on the method in [26], removing parent average effects. The DEBV were then used as response variables to estimate the effect of WFFS status. Weight coefficients ($w_{i}$) were obtained based on [26] using the same DRP package in R.

### Genotyping

DNA from 380 SWB horses was available from our previous study [22]. For all the other horses, DNA was extracted from five to six hair roots in 100 μL of 5% Chelex-100 resin, including 1.4 mg/mL of proteinase K. All DNA samples were genotyped for the XM_001491331: c.2032G>A variant in the *PLOD1* gene by a TaqMan genotyping assay on a StepOnePlus™ instrument (Applied Biosystems, Foster City, CA, USA) by adding 1.5 μL gDNA to TaqMan™ Genotyping Master Mix and Custom TaqMan™ SNP Genotyping Assay (Applied Biosystems, Foster City, CA, USA), for a final volume of 15 µL. The following genotyping parameters were applied: a 10-min hold at 95 °C, followed by 40 cycles of 15 s at 95 °C, and 1 min at 60 °C. Each genotyping plate contained four standard samples: one non-template control, one sample homozygous for the reference allele (Allele 1/Allele 1) and two samples from known WFFS carriers (Allele 1/Allele 2). Two WFFS carrier controls were used to increase the Allele 1/Allele 2 cluster size, since no homozygous individuals for the lethal mutant allele were available.

To characterize the trend in the frequency of WFFS carriers over time, we calculated the frequency of carriers within nine birth year cohorts, starting in 1971 and 2020, and considering a birth interval of 5 years.

### Effect of the WFFS carrier genotype on performance

We estimated the effect of the WFFS carrier genotype on performance using weighted analysis in ASReml 4.1 [27], where the DEBV for the ten traditional evaluating traits and the 50 linear descriptive traits were the response variables. More specifically, in the weighted analysis, the DEBV were the response variables and the $w_{i}$ were the weights calculated as in [26] where the value of $c$ was assumed to be 0.80 following [28]. The EBV were previously estimated using an animal model that included the fixed effects of sex and event (combination of year and place) [23]. Thus, in the weighted model only a birth year period effect (5-years-period) was added to account for possible genetic gain across generations together with the WFFS carrier genotype. Then, we calculated the least square means (LSM) of the WFFS carrier genotypes. Analyses were performed separately for the two subgroups of the SWB breed, which were constituted as in a previous study [12], where horses with EBV for show jumping performance that were higher or lower than the reference population mean of 100 were classified as show jumping (SJ) and non-show jumping (NS) horses, respectively. The large majority of the NS horses could be described as horses bred for the dressage discipline.

### Simulations

In order to model the management of a lethal allele in SWB, we used stochastic genetic simulation to investigate the effect of balancing selection and selection against carriers of a recessive lethal allele, using a population modelled on SWB horses. Simulations were performed with R [25] and the AlphaSimR package [29]. A closed population under selection with a segregating recessive lethal allele was simulated, with the lethal allele either having or not having an effect on the breeding goal traits. The breeding sires were selected for two correlated breeding goals that were simulated as two correlated traits, with the aim of modeling the division of the SWB population into partially overlapping subpopulations selected for show jumping and non-show jumping traits. The correlation between breeding goals was low and positive. As in the SWB population, potential sires were evaluated for both breeding goals, which means that the division of the population was only partial and emerged as a consequence of selection. Forty percent of the simulated sires were selected for Breeding Goal 1, corresponding to non-show jumping traits (traits mainly relevant for dressage). Sixty percent of the sires were selected for Breeding Goal 2, corresponding to show jumping traits. We ran scenarios in which the lethal allele was independent of the breeding goals (no balancing selection) and balancing selection scenarios, in which the lethal allele was also assigned to have a favorable effect on the breeding goals. Each simulation was repeated 100 times. All calculated quantities (for example carrier frequency, genetic values) are presented as averages and 5th and 95th percentiles across replicates.

#### Genetic parameters used in the simulations

The genomes of the founder population were created with the MaCS coalescent simulator, run within the AlphaSimR program [29], using the “GENERIC” population history. This models a generic domestic animal population history, where effective population size decreased gradually from 10^5^ at one million generations ago to 500 at 100 generations ago, and to 100 in the first generation of the simulation. The coalescent simulation resulted in haplotypes of biallelic variants, which were used to form the genomes of the first simulated generation.

We simulated 30 chromosomes of 100 cM, each carrying three randomly chosen causative variants for the breeding goals, which were modelled as two correlated traits with additive genetic effects. The effects of the causative variants on the two breeding goals were drawn from a bivariate normal distribution, with a correlation of 0.3 between the effects on the two breeding goals, which resulted in an average genetic correlation of 0.3 in the first generation (see Additional file 1: Fig. S1). This value is comparable to genetic correlation estimates for dressage and show jumping traits in SWB horses, which vary between trait combinations, but are mostly moderately positive, e.g. 0.32 and 0.45 based on show jumping and dressage traits in two different field tests [30] and from − 0.19 to 0.17 between show jumping traits and dressage competition results [31]. In subsequent simulated generations, the genetic correlation decreased and became, on average, negative as the population was simultaneously selected for the two breeding goals. An increasingly negative genetic correlation is expected from quantitative genetics theory, since variants with positive effects on both breeding goals are fixed more quickly than variants with antagonistic effects [32]. Additional file 1: Fig. S1 shows the change in genetic correlation over generations of simulated selection. Heritability was set to 0.3 for both breeding goals to mimic the level that is commonly estimated for relevant SWB breeding goal traits and used in routine genetic evaluation (Åsa Gelinder Viklund, personal communication). In order to facilitate the comparison, the two breeding goals were scaled to have a mean of 100 and a genetic standard deviation of 20, which is similar to how EBV are scaled in the SWB genetic evaluation.

#### Selection and generation of the next generation

We simulated selection for 20 discrete generations. In each generation, we selected 300 sires out of 3000 male candidates based on phenotypes for the two breeding goals and used all 3000 females as dams. Selection was proportioned so that 120 sires (40%) were selected for Breeding Goal 1 and 180 (60%) were selected for Breeding Goal 2. These proportions are chosen to roughly reflect the proportions of non-show jumping and show jumping horses in the SWB studbook.

The selected sires were randomly mated to the 3000 dams to produce 6000 offspring, with a 50:50 sex ratio. We proportioned the number of offspring to sires based on their phenotype for the breeding goal they were selected on, assigning a larger proportion of the offspring to high-ranking sires. The distribution was based on the proportion of offspring sired by each decile of sires based on the SWB foal statistics from 2008 to 2017 (https://swb.org/betacknings-och-folstatistik/). For example, the decile that sired the smallest number of offspring during this period sired 0.27% of the offspring, while the highest decile sired 58% of the offspring. Based on these proportions, the 6000 offspring were apportioned to deciles of the selected sires, with an equal number of offspring assigned to each sire within the decile. For example, sires in the lowest three deciles were each given one offspring, while sires in the highest decile were each assigned 116 offspring. The whole distribution is shown in Additional file 1: Fig. S2.

#### Recessive lethal allele

The lethal allele was fully recessive and fully penetrant in the simulations. The recessive lethal allele was chosen from variants in the founder population that had a frequency of carriers of ~ 0.10 (between 0.08 and 0.12). In scenarios without balancing selection, we selected the lethal allele at random from 400 neutral variants per chromosome with no effects on either breeding goal. In scenarios with balancing selection, we selected the variant, within the frequency span, that had the largest positive (favorable) effect on Breeding Goal 1 as the lethal allele. The aim was to reflect the associations between WFFS carrier genotypes and the breeding goal traits, that has, a positive effect on non-show jumping traits, but little evidence of negative effects on show jumping traits.

We also ran a simulation scenario for which the genetic effects of the lethal allele were close to the estimates obtained for the WFFS allele. In this case, after selecting the lethal allele as described above, we modified the genetic effect of the lethal allele to be seven units for Breeding goal 1 and zero units for Breeding goal 2. This is similar to our estimate of the association of the WFFS allele with dressage traits and the absence of substantial effects on show jumping traits (see Tables 1 and 2).

**Table 1:** Effect of the WFFS carrier genotype on the ten traditional evaluating traits in the 621 NS horses

Evaluating trait^a^	P-value of WFFS	P-value of birth year cohort	Least square mean (SE) by genotype	
			N/N	WFFS/N
Horse type	NS	**	106.45 (2.14)	110.06 (3.02)
Correctness of legs	NS	*	99.64 (1.08)	100.34 (1.53)
Walk	***	**	110.11 (2.10)	116.19 (2.96)
Trot	*	NS	119.40 (0.89)	124.86 (2.59)
Canter	**	**	107.20 (2.41)	112.77 (3.48)
Jumping technique	NS	NS	80.47 (1.28)	79.25 (1.86)
Jumping temperament	NS	NS	79.43 (1.33)	78.15 (1.93)
Show jumping	*	*	77.85 (1.31)	75.35 (1.80)
Temperament for gait	**	**	112.65 (2.56)	119.30 (3.61)
Dressage	***	***	118.26 (2.56)	125.72 (3.62)

SE: standard error; NS: not significant

*P = 0.05, **0.01 < P < 0.05, ***P < 0.01

^a^EBV in the SWB population have a mean value of 100 and a genetic standard deviation of 20 index units

**Table 2:** Effect of the WFFS carrier genotype on the ten traditional evaluating traits in the 718 SJ horses

Evaluating trait^a^	P-value WFFS	P-value birth year cohort	Least square mean (SE) by genotype	
			N/N	WFFS/N
Horse type	NS	NS	102.97 (1.15)	102.90 (2.56)
Correctness of legs	NS	*	104.35 (0.73)	105.12 (1.62)
Walk	**	NS	94.97 (1.03)	99.42 (2.29)
Trot	NS	NS	94.62 (1.08)	97.86 (2.39)
Canter	NS	NS	105.18 (1.10)	107.56 (2.44)
Jumping technique	NS	NS	125.57 (1.08)	127.00 (2.39)
Jumping temperament	NS	NS	128.81 (2.42)	126.43 (1.09)
Show jumping	NS	NS	128.70 (1.13)	129.13 (2.51)
Temperament for gait	NS	NS	98.24 (1.12)	101.36 (2.47)
Dressage	*	NS	94.10 (1.09)	98.08 (2.22)

LSM: Least square estimation; SE: standard Error; NS: not significant

^*^P = 0.05, ** 0.01 < P < 0.05, ***P < 0.01

^a^EBV in the SWB population have a mean value of 100 and a genetic standard deviation of 20 index units

#### Lethal allele management

We modelled three scenarios of lethal allele management:

Scenario 1: no lethal allele management, reflecting an unknown lethal, for which no genetic testing and management is possible. Affected individuals (homozygous for the lethal allele) were excluded from selection and mating.

Scenario 2: Genetic testing to avoid carrier-carrier mating. Under this scenario, carrier sires were included in the breeding program but could only be mated to non-carrier dams, and vice versa for carrier dams. This is similar to the current policy for known lethal alleles in the SWB studbook. We assumed that genetic testing was free from error.

Scenario 3: Selection against carriers. Under this scenario, carrier sires were excluded from breeding. We varied the intensity of the selection, from strong to weak, by allowing a number (0, 10 or 100) of the top males for each breeding goal to be used regardless of their carrier status. I.e., with 10 top carrier males allowed, any carrier among the top 10 males for each breeding goal were used for breeding but carriers outside the top 10 were removed. This procedure corresponds to allowing up to 6–8% (top 10) or 56–83% (top 100) carriers among the sires used for each breeding goal. All dams were used regardless of their carrier status. The policy of avoiding mating between carriers was maintained, thus the top carrier sires that were used could only be mated to non-carrier dams.

## Results

### Frequency of WFFS carriers

**Fig. 1:**
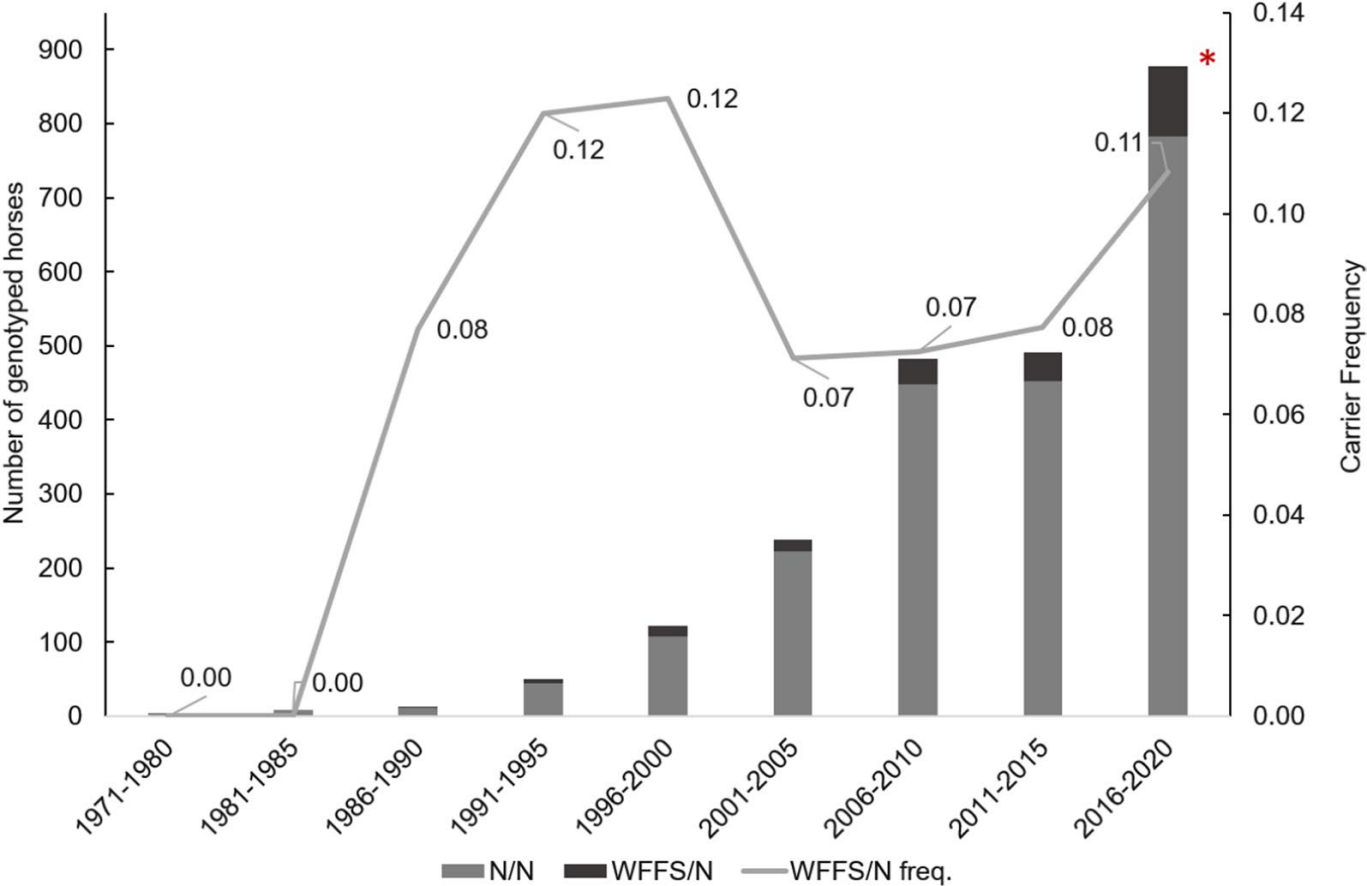
Number of genotyped horses per generation and frequency of WFFS carriers across the nine birth year cohorts. A red asterisk highlights the birth cohort which includes the 511 randomly selected SWB horses

In total, 207 horses (9.0%) among the 2288 genotyped SWB horses were identified as heterozygous for the WFFS mutation (N/WFFS). The remaining 2081 (91.0%) genotyped horses were homozygous for the wild-type allele (N/N). No homozygotes for the WFFS lethal mutation were detected. The number of horses genotyped per birth year cohort varied considerably, the smallest number was observed for the first birth cohort (1971–1980), with five genotyped horses, and the largest for the latest birth cohort (2016–2020), with 878 genotyped horses. No carriers were found in the first birth cohort and the frequency of WFFS carriers was highest (12%) in the fourth and fifth birth cohort, which included horses born between 1991 and 2000 (Fig. 1).

The WFFS carrier frequency calculated from the randomly selected 511 SWB horses born in 2017 was 7.4% (90% confidence interval: 5.3 to 10.0%).

### Effect of the WFFS carrier genotype on performance in NS SWB horses

**Fig. 2:**
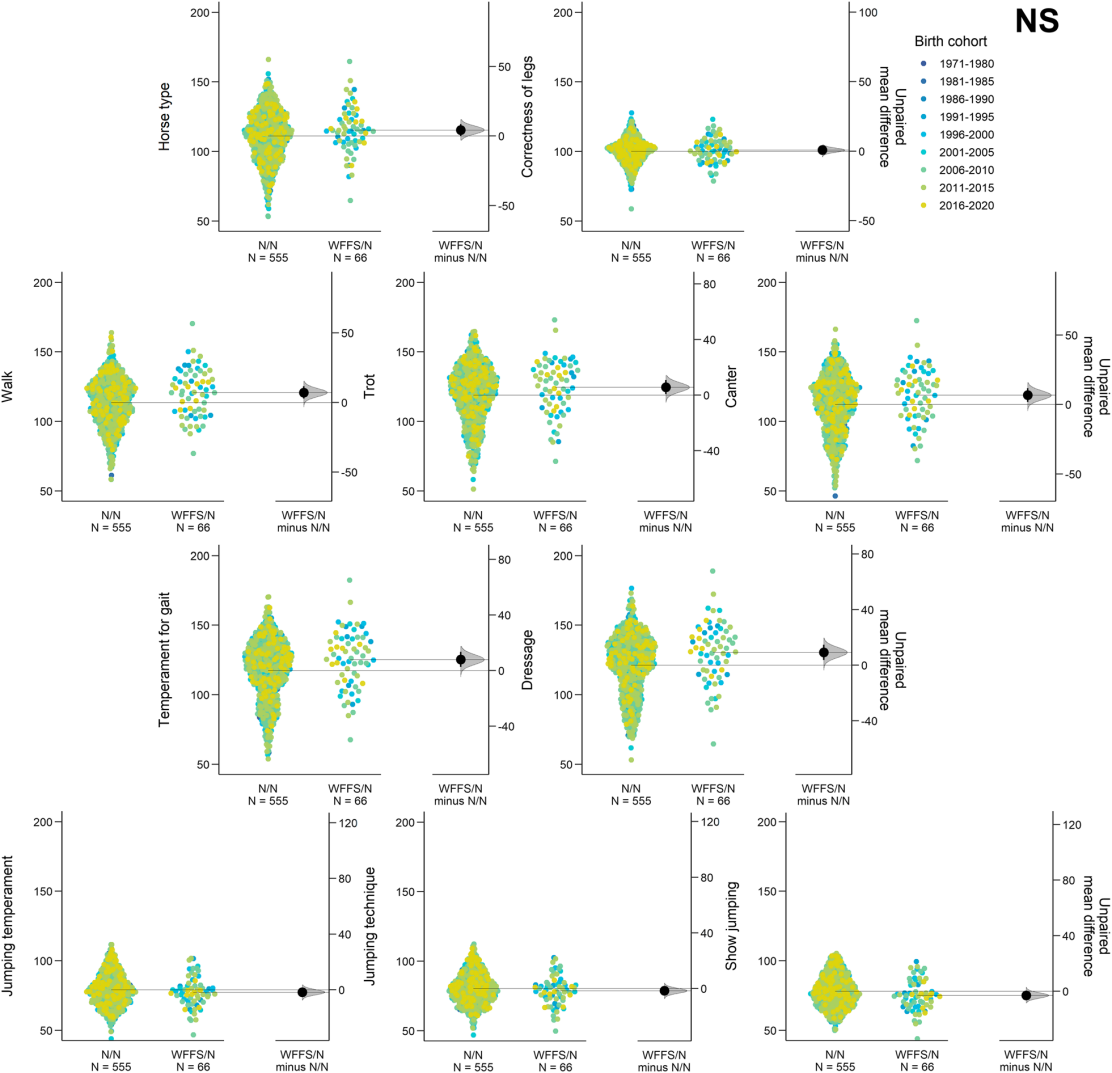
The Gardner-Altman two group estimation plot shows the role of the WFFS carrier genotype on the ten traditional evaluating traits code-colored by the nine birth year cohorts in the NS horses

After removing horses with only PA EBV, 621 NS horses were used to evaluate the effect of WFFS on traditional evaluating traits (see Additional file 2: Table S1). The average reliabilities of EBV for traditional evaluating traits in the NS horses ranged from 0.77 for show jumping to 0.84 for dressage. The frequency of WFFS carriers in the NS horses was 10.6%. The effect of the WFFS carrier genotype was significant for six traditional evaluating traits in the NS horses, with an advantageous effect on the following traditional evaluating traits: the three movement traits (walk, trot, canter), temperament for gaits, dressage ability, and show jumping ability (Table 1 and Fig. 2).

The most advantageous effect of the WFFS allele was found for dressage, with an effect of + 7.46 (+ 0.37 genetic standard deviation) from the comparison of WFFS/N and N/N horses, followed by + 6.95 (+ 0.35 genetic standard deviation) for the trait “temperament for gaits”. In contrast, the horses that were homozygous for the wild-type allele (N/N) scored on average 2.50 higher for show jumping (+ 0.13 genetic standard deviation) compared to horses carrying the WFFS allele (Table 1 and Fig. 2).

**Fig. 3:**
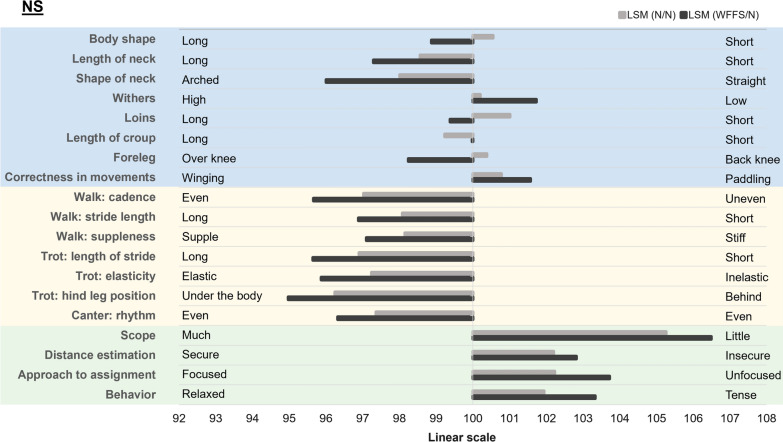
Least square means of the WFFS carrier genotype effect on the 19 significantly affected linear descriptive traits in the NS SWB horses. The conformation traits are highlighted in blue, the gait related traits in yellow and the jumping traits in green

For the linear descriptive traits, 507 NS horses had EBV that were not PA and were included in the analyses. The average reliabilities for those traits ranged from 0.61 to 0.81. Of the 50 linear descriptive traits, 19 were significantly affected by the WFFS carrier genotype in the NS horses (see Additional file 3: Table S2). Overall, according to the linear description, WFFS/N NS horses had a longer body, neck, and loins compared to N/N horses. They also had a more arched neck, lower withers, a shorter croup, the front legs were more over the knee, and had more paddling movements. Compared to N/N horses, WFFS/N NS horses had a more even walk in terms of cadence, were more supple, and with a longer stride, their trot was more elastic with a longer stride, their hind leg was positioned more under the body, and they had a more even rhythm in canter. When jumping, WFFS/N NS horses had lower scope, were less focused, less secure in the distance estimation, and more tense than N/N NS horses (Fig. 3).

### Effect of the WFFS carrier genotype on performance in SJ SWB horses

**Fig. 4:**
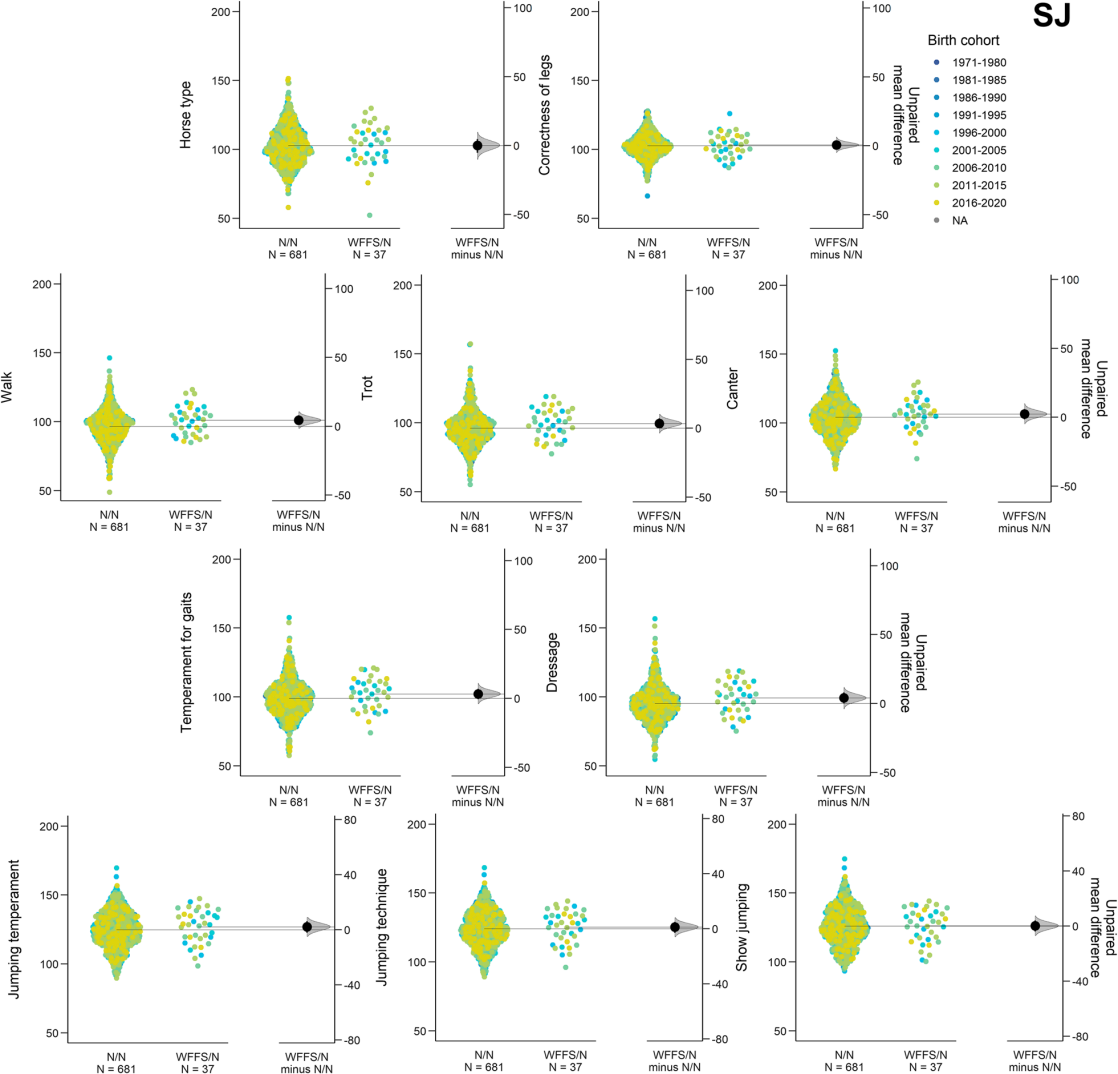
The Gardner-Altman two group estimation plot shows the role of the WFFS carrier genotype on the ten traditional evaluating traits code-colored by the nine birth year cohorts in the SJ horses

After removing horses with only PA EBV, 718 SJ horses were included to evaluate the effect of WFFS on traditional evaluating traits (see Additional file 4: Table S3). The average reliabilities of EBV for the traditional evaluating traits in the NS horses ranged from 0.74 for dressage to 0.79 for show jumping EBV. The WFFS carrier frequency in the SJ horses was equal to 5.1%. The effect of the WFFS carrier genotype was significant for two traditional evaluating traits in the SJ horses: walk and dressage (Table 2, and Fig. 4).

The most advantageous effect of the WFFS allele was found for the walk trait, with a difference equal to + 4.48 (+ 0.22 genetic standard deviation) between WFFS/N and N/N horses, followed by + 3.98 (+ 0.20 genetic standard deviation) for dressage (Table 2, and Fig. 4).

**Fig. 5:**
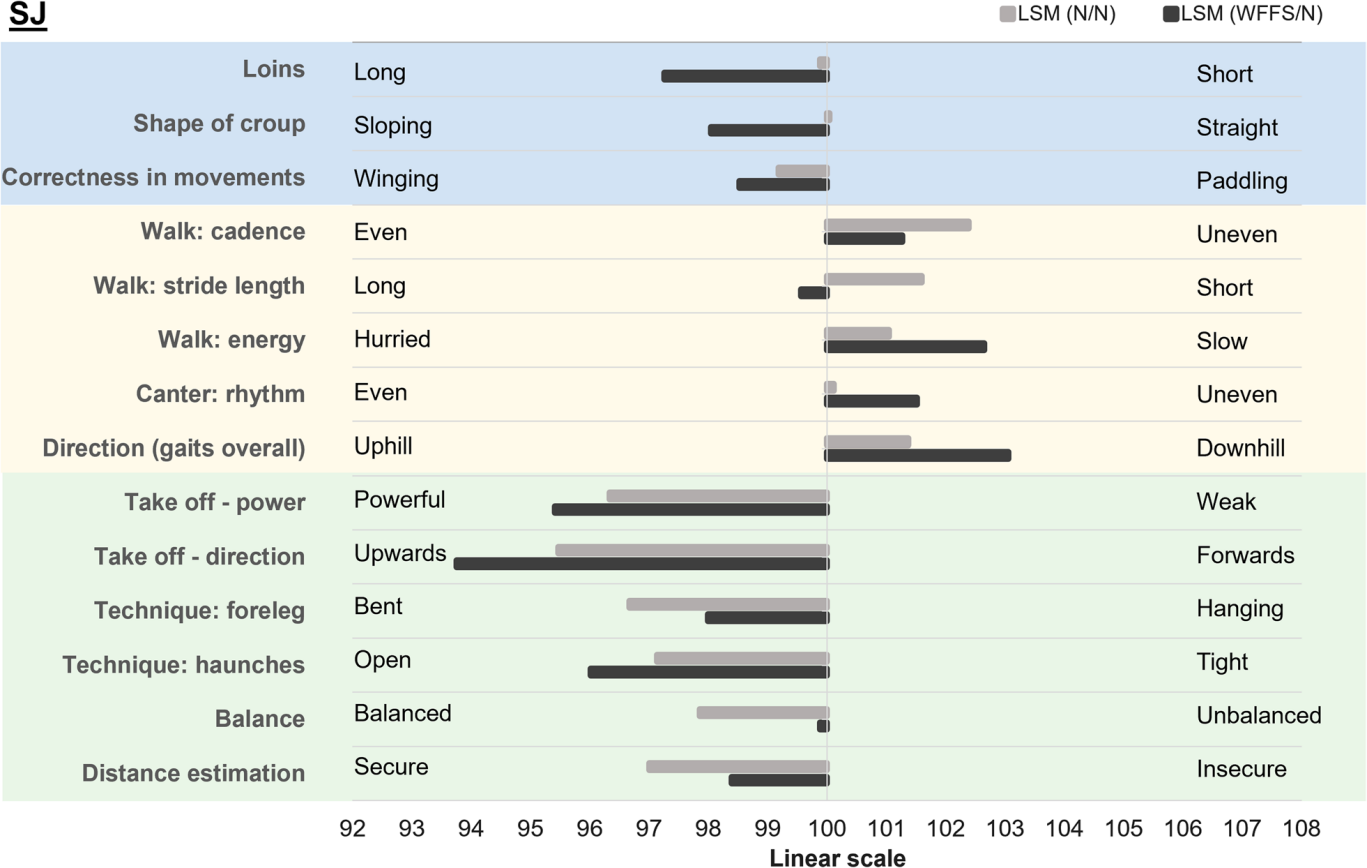
Least square means of the WFFS carrier genotype effect on the 14 significantly affected linear descriptive traits in the SJ SWB horses. The conformation traits are highlighted in blue, the gait related traits in yellow and the jumping traits in green

For the linear descriptive traits, 569 SJ horses fulfilled the reliability filter and were included in further analyses. The average reliabilities of the EBV for those traits ranged from 0.62 to 0.80. Of the 50 linear descriptive traits, 14 traits were significantly affected by the WFFS carrier genotype in the SJ horses (see Additional file 3: Table S2). According to the linear description, WFFS/N SJ horses had longer loins, a more sloping croup, and more winging movements than N/N horses. The walk in WFFS/N SJ horses was longer, more even in terms of cadence, less energetic, and with a more downhill general gait direction compared to N/N horses. The canter had a more uneven rhythm in WFFS/N than in N/N horses. For jumping traits, WFFS carrier SJ horses showed a more powerful jump with upwards take-off, with more hanging front legs, more open haunches, and less balance and more insecure distance estimation than N/N horses (Fig. 5).

### Simulation study of balancing selection and recessive lethal management

#### Effect of balancing selection

**Fig. 6:**
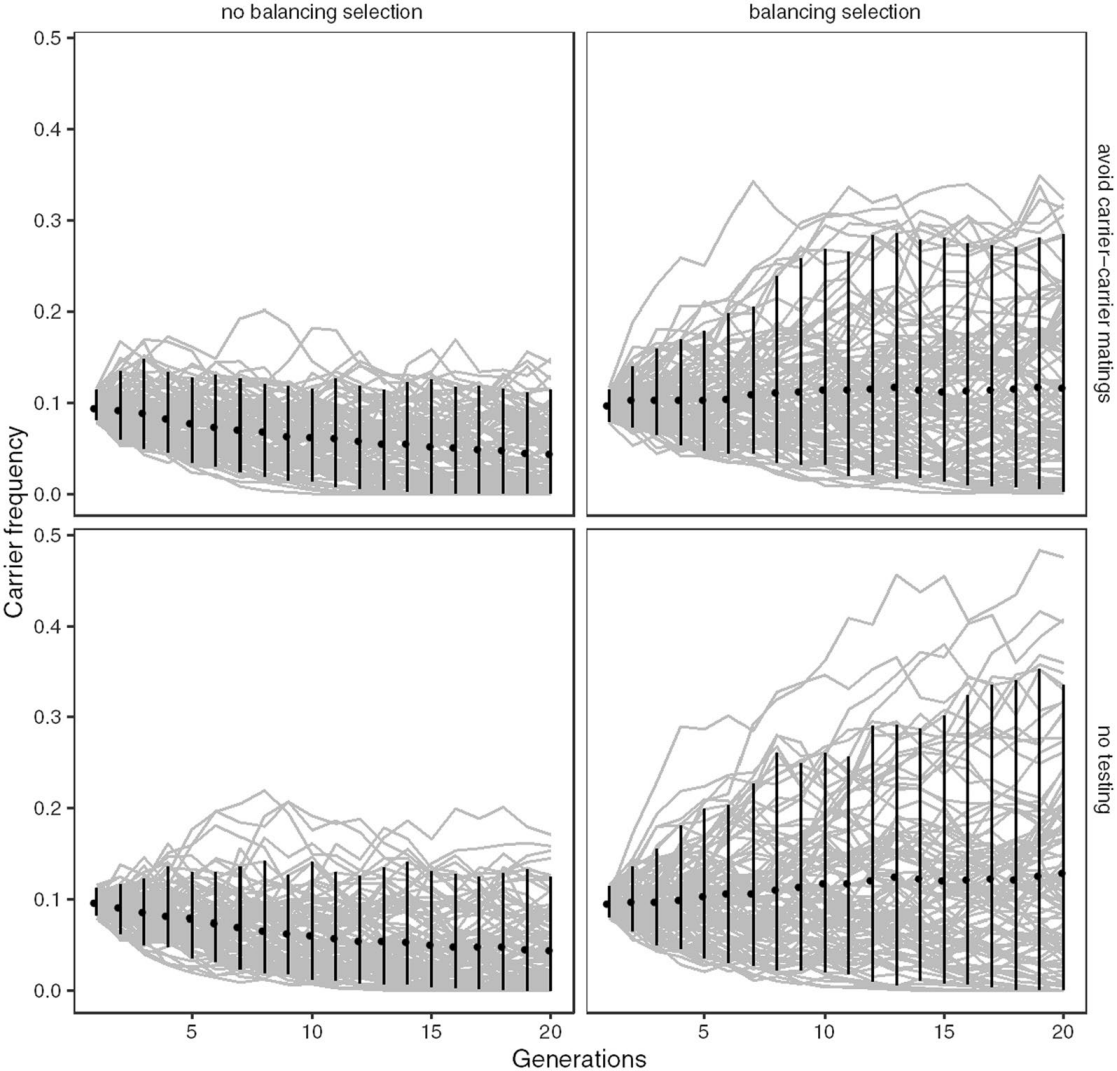
Frequency of the carriers of the recessive lethal allele in simulated breeding programs. Frequency of carriers over 20 generations of breeding without (panels to the left) and with balancing selection (panels to the right), in scenarios where genetic testing is used to avoid mating between carriers (top panels) or in a scenario where the lethal allele is unknown, and no testing is available (bottom panels). The points show the mean frequency of carriers over simulation replicates, with error bars showing the 5 and 95 percentiles, and the grey lines in the background showing the individual simulation replicates

The simulations showed that balancing selection resulted in higher frequencies of carriers. Figure 6 shows the effect of balancing selection in a scenario when genetic testing is used to avoid mating between carriers, and when the lethal allele is unknown and there is no testing. In the absence of balancing selection, the frequency of carriers on average decreased over time (from the starting point of 10%), but very slowly and with substantial random variation. At the end of the simulation, the mean frequency of carriers without balancing selection was 4.4% (standard deviation ± 3.9%) with genetic testing and 4.5% (standard deviation ± 4.1%) without. With balancing selection, on average the frequency of carriers remained high and the random variation was greater, leading to very high (above 30%) frequencies of carriers in some simulation replicates. At the end of the simulation with balancing selection, the average carrier frequency was 12% (standard deviation ± 8.8%) with genetic testing and 13% (standard deviation ± 11%) without. Thus, when genetic testing was available to avoid mating between carriers, the frequency of carriers was slightly lower.

#### Selection against carrier sires

**Fig. 7:**
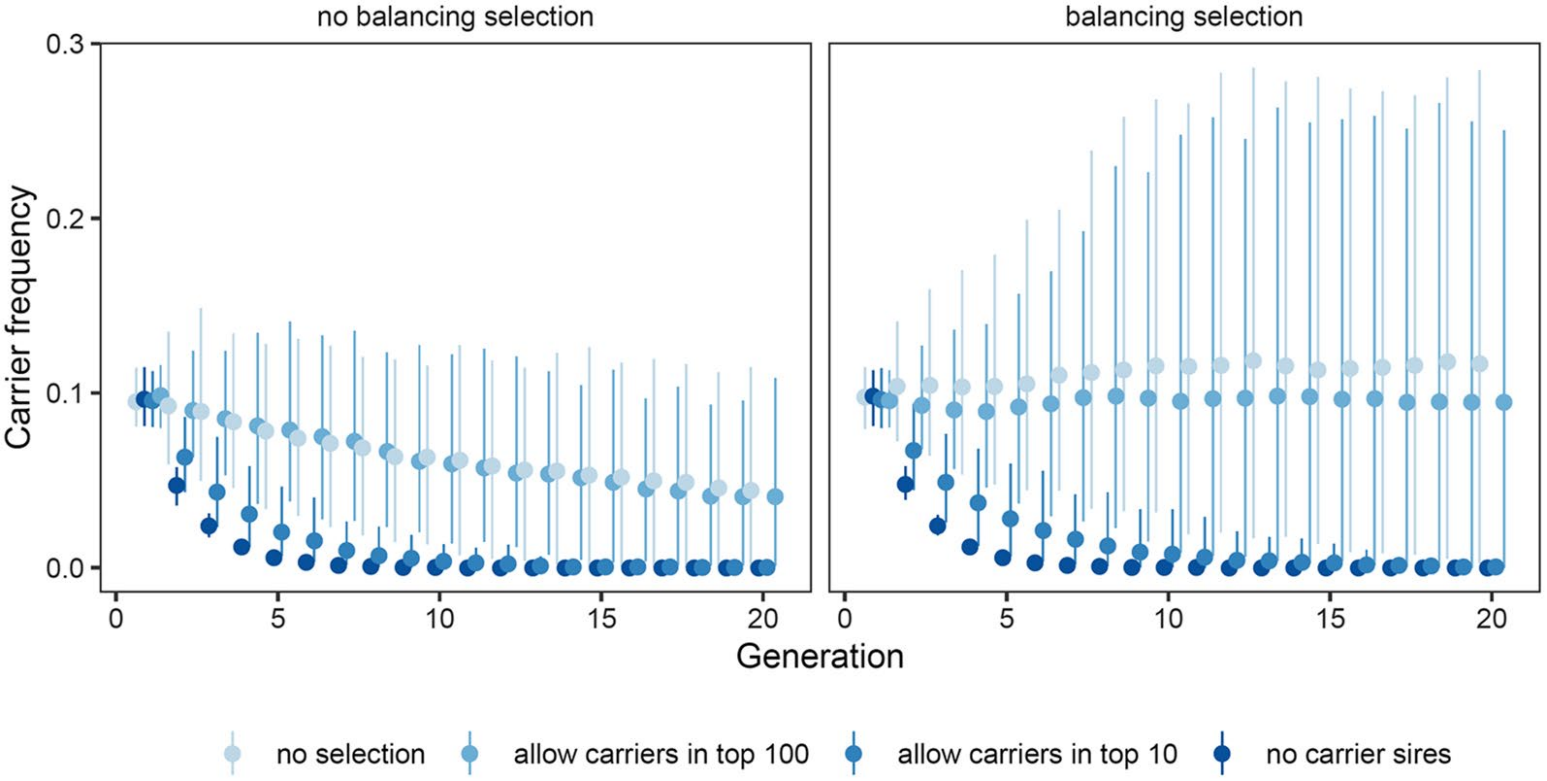
Effect of selection against the lethal allele. The average frequency of carriers over simulation replicates, with error bars reflecting the 5 and 95 percentiles. The scenarios are (1) complete selection against carrier sires (i.e., all carrier sires are excluded), and (2) when the carriers among the sires in either the 10 or top 100 of each breeding goal are included regardless of their carrier status

The results showed that selection against carrier sires can substantially reduce the frequency of the lethal allele, even when not all carrier sires are excluded (Fig. 7). On average, the frequency of carriers decreased to less than 1% within four generations when all carrier stallions were excluded from breeding. We varied the strength of selection against the lethal allele by allowing exemptions for carrier sires with particularly high EBV (those in the top 10 or the top 100 for either breeding goal). Even when carrier sires that were in the top 10 of either breeding goal were allowed to breed, the frequency of carriers still decreased to less than 1% within four generations. However, when carrier sires that were in the top 100 of either breeding goal were allowed to breed, the decrease in carrier frequency was substantially slowed down, with the carrier frequency still around 8 and 9% without and with balancing selection, respectively, after four generations. Balancing selection greatly increased the random variation in frequency of carriers when many carrier sires were allowed to breed.

Selection against carrier sires did not affect the genetic progress for the breeding goal much. There was little difference between the average genetic mean of the population after 5, 10, 15 or 20 generations with different selection emphasis against carrier sires, but substantial random variation (see Additional file 1: Fig. S3).

#### Subpopulation differences between the two breeding goals

**Fig. 8:**
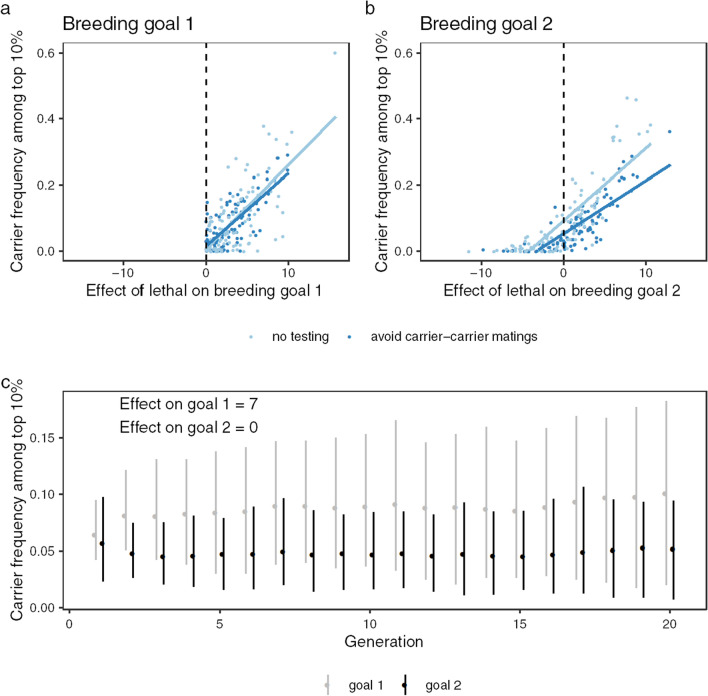
Relationship of the effect of the simulated lethal allele on the breeding goals with the carrier frequency. Shown is the positive relationship of the frequency of carriers among the top 10% individuals at the end of the simulation (generation 20) with the additive effect of the lethal allele on (**a**) breeding goal 1 and (**b**) breeding goal 2, and (**c**) shows the top 10% carrier frequencies for breeding goal 1 and breeding goal 2 in each generation in simulations where the effect on breeding goal 1 was seven units and the effect on breeding goal 2 was zero, in line with our estimates from the SWB population. The dashed line in (**a**) and (**b**) indicates a genetic effect of zero

Balancing selection that affects the two breeding goals differently can drive to relatively small differences in the frequency of carriers between subpopulations. Figure 8a and b show the relationship of the effect on each breeding goal with the subpopulation frequencies of carriers (frequency of carriers among the top 10% individuals for each breeding goal), with a positive relationship between the effect on the breeding goal and the frequency of carriers. Figure 8c shows the results of a simulation scenario with genetic parameters chosen such that they are close to the estimates obtained for the genetic effects of the WFFS allele (an effect of seven units on Breeding goal 1 and no effect on Breeding goal 2). In this case, the frequency of carriers among the top 10% individuals was higher for Breeding goal 1 than for Breeding goal 2. At generation 20, the average difference was 4.9% (with 5 and 95 percentile limits from 0.7 to 10%).

## Discussion

In this study, we estimated the frequency of carriers of the WFFS allele in the SWB breed and found that the WFFS allele is associated with advantageous effects on several performance traits, especially those related to movements, which was more marked in the NS SWB horses. This might be indicative of balancing selection on the WFFS allele in the case of dressage-related traits. Using simulation, we showed: (1) how balancing selection can maintain a recessive lethal allele in a population with similar features to the SWB breed, (2) that the decline in frequency of the lethal allele in the absence of balancing selection is expected to be slow, and (3) that selection against carrier sires can result in a more rapid reduction of the frequency of the mutant allele.

In the light of these results, we discuss the frequency of WFFS in SWB horses compared to frequency estimates in other horse breeds, propose some evidence for balancing selection on WFFS, and discuss the potential effect of testing and selection on carrier frequency, thereby evaluating the implications for breeding practice.

### Carrier frequency of WFFS in Swedish warmbloods

Our estimate of the frequency of WFFS carriers among the randomly selected samples (7.4%) was lower than estimates obtained in German and Brazilian Warmbloods [6, 7], and across Warmblood breeds [8, 10]; however, the two latter studies found no WFFS carriers among the small number of 18 SWB horses sampled.

Random sampling from the population is fundamental when estimating carrier frequencies. Therefore, we recommend that estimates of frequencies based on non-random samples, such as the 12% frequency in SWB horses between 1991 and 2000 in our study, are interpreted with caution. These data clearly show that the WFFS allele was present in the SWB population, already in the middle 1980s, but the estimate of the frequency may be biased.

The apparent discrepancy between 0% WFFS carriers found in 16 SWB [8] and 7.4% in our study, is not significant and is likely due to sampling errors. If we assume random sampling in a population with a frequency of carriers of 7.4%, there is a 29% probability of drawing only non-carriers with a sample size of 16 (from the probability mass function of the binomial distribution), and thus, the 0% estimated frequency of carriers is not significantly different from our estimate (Fisher’s exact test comparing 0 out of 16, to 38 out of 511, p = 0.62). Thus, the two studies are not statistically different.

### Support for balancing selection on the WFFS allele

The association between WFFS carrier genotype and dressage-related traits suggests the potential presence of balancing selection, where natural selection against the WFFS recessive lethal allele is counteracted by its apparent advantageous effects, especially for traits related to movement and conformation. Our study further supports this positive effect of the WFFS allele on the linear descriptive traits of walk, trot and canter cadence, stride length, suppleness, and elasticity. These results were more evident in the case of NS horses, which are mainly selected for the dressage discipline. This could be explained by a stronger selection for high mobility in dressage horses, with extreme locomotion patterns, possibly as a result of impaired collagen structure due to a lower LH1 enzyme activity in carriers of the mutant *PLOD1* allele. In humans, a LH1 activity of 52% was measured in carriers of a similar *PLOD1* variant (c.2032G>A, p.Gly678Arg) as in the equine lethal WFFS allele, which is consistent with an intermediate phenotype, plausibly presenting a higher degree of joint laxity [33]. Likewise, a high prevalence of carriers of variants of connective tissue genes were found in professional ballet dancers, possibly accounting for the high degree of mobility in this population [34]. Interestingly, the walk seemed to be the most affected gait by WFFS carrier status, both from the traditional evaluating and linear descriptive evaluations in the NS and SJ subgroups. In addition, some linear descriptive conformation traits were significantly affected by the WFFS status. Loins were significantly longer in WFFS carriers for both NS and SJ subgroups than in N/N horses. Likewise, a previous study in horses found a significant effect of WFFS carrier status on conformation traits, such as body quality, head, neck, frame, saddle area, front and hindlimbs [6]. However, the relevance of the WFFS allele for conformation remains to be justified.

EBV are not only estimated based on an individual’s own performance but include information from relatives, which could add uncertainty to the attributions of genetic effects to any specific genotyped locus. To overcome this, we excluded all the horses that had EBV relying on parental average only and used as response variable the de-regressed EBV to weight for differences in reliabilities and to account for heterogeneous variance.

Our simulations showed that allele frequencies are not fully informative for balancing selection. At all time-points, there was a considerable overlap between frequencies of carriers in scenarios with and without balancing selection, which means that the presence of balancing selection cannot be inferred from the frequency of the WFFS allele alone.

Nevertheless, in the simulations, differences in frequencies of carriers between the top individuals for the two breeding goals (representing selection for non-show jumping and show jumping horses) tended to occur only with balancing selection. In the absence of balancing selection, the lethal allele can still drift to reach different frequencies in the two subpopulations. However, on the one hand, genetic drift will be symmetrical, in that there is no bias towards a higher frequency in any one subpopulation. On the other hand, selection will drive the lethal allele towards a higher frequency in the subpopulation where there is a beneficial effect of the lethal allele on the breeding goal; the size of the difference will depend on the size of the effect. In the real data, we found that the frequency of WFFS carriers was almost four times higher in the top 10% NS horses than in the top 10% SJ horses (17.2% compared to 4.4%), which strengthens the hypothesis of balancing selection for carriers, especially in dressage horses. Thus, taken together, the differences in frequency of carriers between subpopulations of Swedish Warmblood horses, and the observation that the WFFS allele has a similarly high frequency across Warmblood breeds [6–8], are suggestive of balancing selection for the WFFS allele in these populations, especially for gait-related traits.

### Potential effects of testing and selection against carrier sires

While the policy of using genetic testing for WFFS to avoid mating between carriers to eliminate the risk of affected foals being born is beneficial for animal welfare, simulations showed that such testing and mating strategies are not expected to decrease the frequency of carriers over time. If, instead, the aim is to decrease or eliminate the WFFS allele in the population, selection against carriers would be needed because the decline of such a recessive lethal allele is slow, even in the absence of balancing selection, with the allele still persisting in the simulated populations at a frequency of ~ 5% after 20 generations.

Selection against carrier sires led to a decline of the lethal allele, decreasing the frequency of carriers to less than 1% within four generations. Selection against carrier sires does not need to be complete, since allowing a few carrier sires to be included in the breeding program, did not make much difference in the rate of decline of the frequency of carriers. However, in practice, the effect of allowing carrier sires will also depend on to what extent they are used in breeding. Simulations have shown that the use of popular sires increases the risk of lethal alleles becoming frequent in a population [35]. Thus, an alternative suggestion could be to limit the number of mares that are mated to WFFS carrier sires.

### Implications for breeding

All in all, our results suggest that one course of action for reducing the frequency of WFFS carriers, without losing the gain in breeding goal traits, is to implement marker-assisted selection against at least a fraction of the carrier sires. Intuitively, this is possible when the excluded carrier sires are replaced by non-carrier sires that are almost as good in terms of their EBV for the breeding goal traits. However, this would be different when multiple known genetic defects have to be taken into account. With multiple defects, requiring sires to be non-carriers for all defects could substantially reduce the number of eligible males, resulting in compromised genetic gain and increased inbreeding. Thus, as Georges et al. [36] have argued, when there are many genetic defects that need to be balanced, culling all carrier sires is not the right approach. To address this, several mate selection schemes have been devised that could be used to balance genetic gain with selection against genetic defects [37, 38]. In principle, such schemes are conceptually similar to the simple selection system with exemptions for high-ranking sires that we proposed: carrier males would be penalized in the evaluation, but they could still be used, in matings with non-carrier dams, if they have high EBV; the penalties could be derived from economic calculations, and mate selection could involve other aspects such as inbreeding.

Given that the generation interval of SWB is 10 to 11 years [23], testing and avoiding mating between carriers will have to be maintained at least for decades to eradicate the lethal WFFS allele. Our results suggest, but do not conclusively prove, the presence of balancing selection on the WFFS allele. If there is balancing selection, the frequency of carriers is likely to remain the same or to increase. If not, the frequency of carriers is likely to decrease slowly. Thus, in both cases, selection against carrier sires would be needed to reliably decrease the frequency of carriers. If only the top performing carrier breeding stallions are used, the genetic gain could still be maintained.

## Conclusions

This work revealed several significant associations of the *PLOD1* c.2032G>A, (p.Gly678Arg) WFFS causative missense variant with performance traits in SWB horses, and in particular, highlighted a positive effect on movement-related traits. We also detected an association with conformation traits, since the carriers of the WFFS lethal allele had longer body conformation. Our findings suggest the potential presence of balancing selection for the WFFS allele in SWB horses that are bred for dressage purposes. However, further research is needed to confirm the apparent association of genes related to collagen deficiencies and joint laxity syndromes with equine performance. Using simulations, we showed that by selecting only carrier stallions if they are in the top (10%) for the breeding goal, the frequency of the deleterious allele would decrease substantially in a few generations, while maintaining genetic gain. In the future, we will aim at gaining more knowledge about performance traits under selection in sport horses by identifying associated genetic markers or novel causative mutations which might serve as new tools in horse breeding to select for healthy, sustainable, and better performing horses.

## Supplementary Information

**Additional file 1: Figure S1.** Decline in genetic correlation between the two breeding goal traits over generations of selection. The figure shows the genetic correlation between breeding goals in a scenario without balancing selection and avoiding carrier—carrier matings. Points show the average over replicates, with the error bars being the 5 and 95 percentiles. **Figure S2.** Distribution of offspring per sire used in the simulation. The figure shows the number of offspring per sire proportioned to sires in that decile. **Figure S3.** Balancing selection on the lethal allele has little effect on genetic gain in the simulation. The figure shows the average genetic values for simulated populations at generations 1, 5, 10, 15 and 20 for the two breeding goals, with error bars showing the 5 and 95 percentiles.

**Additional file 2: Table S1.** Descriptive statistics of the traditional evaluating traits in the NS horses. The following values are reported for each traditional evaluating trait in the NS horses: number of observations, mean, standard deviation, minimum, 25th percentile, 75th percentile and maximum.

**Additional file 3: Table S2.** Effect of the WFFS genotype on the DEBV for the linear descriptive traits for both NS and SJ SWB horses. Table S2 shows the effect of the WFFS expressed in significance level and in LSM of the DEBV for the linear descriptive traits per each subgroup (NS and SJ SWB). EBV for the linear descriptive traits in the SWB population have a mean value of 100 and a genetic standard deviation of 4 index units.

**Additional file 4: Table S3.** Descriptive statistics of the traditional evaluating traits in the SJ horses. The following values are reported for each traditional evaluating trait in the SJ horses: number of observations, mean, standard deviation, minimum, 25th percentile, 75th percentile and maximum.

## Data Availability

The datasets in the current study were generated and analyzed in collaboration with the Swedish Warmblood Association, and have a commercial value for them. Thus, the SWB horse data are available from the corresponding author on reasonable request. The simulation scripts are available at: https://github.com/mrtnj/WFFS.
