# Supplementary material for: Performance of Swedish Warmblood fragile foal syndrome carriers and breeding prospects
Source: Genet Sel Evol. 2022 Jan 21;54:4. doi: 10.1186/s12711-021-00693-4 (PMC8783495; doi:10.1186/s12711-021-00693-4)
Supplement: Supplementary file 1 — Additional file 1: Figure S1. Decline in genetic correlation between the two breeding goal traits over generations of selection. The figure shows the genetic correlation between breeding goals in a scenario without balancing selection and avoiding carrier—carrier matings. Points show the average over replicates, with the error bars being the 5 and 95 percentiles. Figure S2. Distribution of offspring per sire used in the simulation. The figure shows the number of offspring per sire proportioned to sires in that decile. Figure S3. Balancing selection on the lethal allele has little effect on genetic gain in the simulation. The figure shows the average genetic values for simulated populations at generations 1, 5, 10, 15 and 20 for the two breeding goals, with error bars showing the 5 and 95 percentiles. [file 12711_2021_693_MOESM1_ESM.docx]

**Figure S1. Decline in genetic correlation between the two breeding goal traits over generations of selection. The figure shows the genetic correlation between breeding goals in a scenario without balancing selection and avoiding carrier—carrier matings. Points show the average over replicates, with the error bars being the 5 and 95 percentiles.**

**Figure S2. Distribution of offspring per sire used in the simulation. The figure shows the number of offspring per sire proportioned to sires in that decile.**

**Figure S3. Balancing selection on the lethal allele has little effect on genetic gain in simulation. The figure shows average genetic values for simulated populations at generation 1, 5, 10, 15 and 20 for the two breeding goals, with error bars showing the 5 and 95 percentiles.**
